# AI-ASSISTED METHODS FOR ASSESSING AFFECT AND BEHAVIORAL SYMPTOMS IN DEMENTIA: A SYSTEMATIC REVIEW

**DOI:** 10.1093/geroni/igac059.2774

**Published:** 2022-12-20

**Authors:** Ying-Ling Jao, Yo-Jen Liao, Fengpei Yuan, Ziming Liu, Xiaopeng Zhao, Wen Liu, Diane Berish, James Wang

**Affiliations:** Pennsylvania State University, University Park, Pennsylvania, United States; Pennsylvania State University, State College, Pennsylvania, United States; University of Tennessee, Knoxville, Tennessee, United States; University of Tennessee, Knoxville, Tennessee, United States; University of Tennessee, Knoxville, Tennessee, United States; University of Iowa, Iowa City, Iowa, United States; Pennsylvania State University, University Park, Pennsylvania, United States; Pennsylvania State University, University Park, Pennsylvania, United States

## Abstract

Negative affect and neurobehavioral symptoms occur in most people with dementia and significantly impact their health outcomes and sense of wellbeing. Detecting these symptoms in this population is challenging due to associated cognitive impairment and communication difficulties. Innovative technology and artificial intelligence (AI)-assisted tools are emerging for assessing affect and neurobehavioral symptoms in individuals with dementia. This review synthesizes research evidence to identify existing AI-assisted measurement tools and evaluate their accuracy in assessing affect and symptoms in people with mild cognitive impairment and dementia. PubMed, CINAHL, Scopus, and Web of Science databases were searched. Eight articles were identified. Multiple machine learning (ML) models were developed to assess affect, apathy, anxiety, depression, agitation, and wandering. One ML model detected positive and negative affect via facial expression with an overall accuracy of 86%. One ML model detected apathy based on speech and achieved an area under curve (AUC) accuracy of 0.77–0.88. Another speech-based ML model, based on paralinguistic markers, predicted apathy, anxiety, and depression by ≥0.3 points. Another model detected wandering based on activity monitoring data and showed 98% sensitivity and specificity. Furthermore, multiple ML models were developed to detect agitation using multi-modal sensors with AUC ranging from 0.50–0.82. Findings suggest that AI-assisted tools are a promising approach to detecting affect and neurobehavioral symptoms, yet the evidence is limited. More research is needed to develop comprehensive, accurate models to detect neurobehavior symptoms. The results have significant implications for supporting research and clinical practice to promote quality of care for people with dementia.

